# *Neurog2* regulates *Isl1* to modulate horizontal cell number

**DOI:** 10.1242/dev.201315

**Published:** 2023-01-05

**Authors:** Patrick W. Keeley, Pooja S. Patel, Matthew S. Ryu, Benjamin E. Reese

**Affiliations:** ^1^ Neuroscience Research Institute, University of California at Santa Barbara, Santa Barbara, CA 93106-5060, USA; ^2^ Department of Psychological and Brain Sciences, University of California at Santa Barbara, Santa Barbara, CA 93106-5060, USA

**Keywords:** Epistasis, QTL, SNP, Variant, E-box, Recombinant inbred strain, Conditional knockout

## Abstract

The population sizes of different retinal cell types vary between different strains of mice, and that variation can be mapped to genomic loci in order to identify its polygenic origin. In some cases, controlling genes act independently, whereas in other instances, they exhibit epistasis. Here, we identify an epistatic interaction revealed through the mapping of quantitative trait loci from a panel of recombinant inbred strains of mice. The population of retinal horizontal cells exhibits a twofold variation in number, mapping to quantitative trait loci on chromosomes 3 and 13, where these loci are shown to interact epistatically. We identify a prospective genetic interaction underlying this, mediated by the bHLH transcription factor *Neurog2*, at the chromosome 3 locus, functioning to repress the LIM homeodomain transcription factor *Isl1*, at the chromosome 13 locus. Using single and double conditional knockout mice, we confirm the countervailing actions of each gene, and validate *in vitro* a crucial role for two single nucleotide polymorphisms in the 5′UTR of *Isl1*, one of which yields a novel E-box, mediating the repressive action of *Neurog2*.

## Teaser

**Summary:** Quantitative trait loci mapping identifies two genomic loci that epistatically modulate horizontal cell number in the mouse retina, uncovering a novel interaction between *Neurog2* and *Isl1* in the development of these cells.

## INTRODUCTION

The determination of neuronal number is controlled by various developmental processes, including proliferation, fate determination, differentiation and cell death. The retina has proven an invaluable model for studying how these processes shape the sizes of neuronal populations, and for exploring the genetic mechanisms underlying those events. In the mouse retina, for instance, variability in the sizes of different cellular populations within any inbred strain is low, indicative of the precision by which cell number of achieved, yet there is considerable variability in the size of any population between different inbred strains (Williams et al., 1998b; Keeley et al., 2014), owing to multiple genetic variants that modify those developmental processes (Kautzman et al., 2018a,b; Whitney et al., 2009, 2011a,b; Williams et al., 1998a). Polygenic traits such as these may arise through the actions of genetic variants that independently modulate cell number, or epistatically, through a genetic interaction. Here, we identify a prospective epistatic interaction controlling the size of one cellular population within the retina, the horizontal cells.

Retinal horizontal cells show a nearly twofold variation in their number when comparing different laboratory strains of mice (Whitney et al., 2011a; Williams et al., 1998a). The genomic sources of that variation can be identified by exploring recombinant inbred (RI) strains of mice, mapping phenotypic variation across the strains to controlling genomic loci. Using such a forward-genetic approach, we previously identified a quantitative trait locus (QTL) at the distal tip of chromosome (Chr) 13, accounting for approximately 40% of the variation in horizontal cell number (Whitney et al., 2011a). We identified a candidate causal gene at this locus, *Isl1*, subsequently confirming that loss of ISL1 function led to a significant increase in the horizontal cell population. Microarray analysis of *Isl1* gene expression across the RI strains in maturity revealed a negative correlation with horizontal cell number, and qPCR analysis during the period of horizontal cell production confirmed significant differences in *Isl1* transcript levels between the two originating parental strains of the RI strain-set, A/J and C57BL/6J (B6/J), the strain with 50% fewer horizontal cells exhibiting significantly greater *Isl1* expression. Finally, we identified a candidate causal genetic variant within *Isl1* as a single nucleotide polymorphism (SNP) creating a novel E-box within the 5′UTR (Whitney et al., 2011a). We hypothesized that the presence of this E-box enables the suppression of *Isl1*, in turn raising horizontal cell number.

Here, we explore the subsidiary variation in horizontal cell number beyond that accounted for by the QTL at the *Isl1* locus on Chr 13. Using composite interval mapping, we identify a second prospective controlling locus on Chr 3. By separating the RI strains by their haplotypes at these two Chrs 3 and 13 loci, we show evidence for an epistatic interaction controlling horizontal cell number as well as *Isl1* expression across the strains. Using bioinformatic resources, we parse the Chr 3 QTL, identifying a prospective candidate gene, neurogenin 2 (*Neurog2*), a proneural basic helix-loop-helix (bHLH) transcription factor known to bind the E-box. We find that a loss of NEUROG2 function yields a significant reduction in the horizontal cell population. Interestingly, *Neurog2* expression levels correlate positively with horizontal cell number across the RI strains, but only amongst those strains containing the novel E-box; furthermore, we demonstrate, using an *in vitro* luciferase assay, that there is a progressive decline in the ability of the *Isl1* 5′UTR to activate luciferase as a function of increasing *Neurog2* expression, provided that the *Isl1* regulatory region contains the B6/J sequence, which includes the E-box. As we hypothesize that *Neurog2* modulates horizontal cell number upstream of *Isl1* via this E-box, we demonstrate in the *Isl1/Neurog2* double conditional knockout (DCKO) retina that the decline in horizontal cell number produced by loss of NEUROG2 function is abrogated, with horizontal cell number increasing, as observed following loss of only *Isl1*, confirming the genetic interaction between these two genes in regulating the size of the horizontal cell population.

## RESULTS

### A second QTL on Chr 3 modulates horizontal cell number

**Fig. 1.**
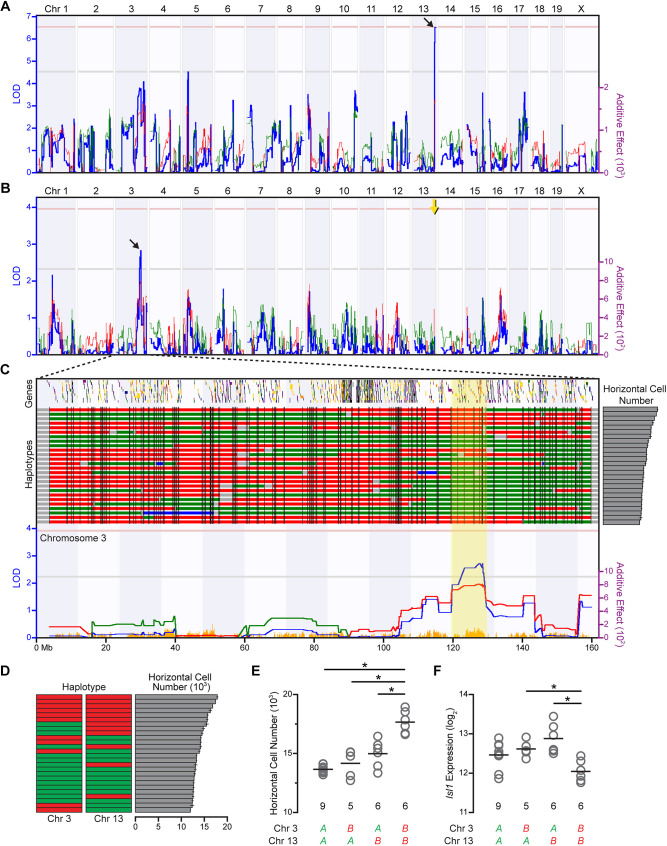
**QTL mapping reveals an interaction between Chrs 3 and 13 in modulating horizontal cell number.** (A) Simple interval mapping of the variation in horizontal cell number across 26 RI strains of the AXB/BXA strain set had previously revealed a significant QTL on Chr 13 (diagonal black arrow). The blue trace indicates the LOD score, being an index of the strength of association between phenotype and genotype across the genome (left *y*-axis). The green and red traces plot the additive effect of the presence of the *A* versus *B* haplotype, respectively, upon increasing horizontal cell number (right *y*-axis). Horizontal dashed pink and gray lines indicate significant (*P*<0.05) and suggestive (*P*<0.67) thresholds, respectively, determined by permutation testing. (B) Using composite interval mapping to control for the variation in cell number attributed to the Chr 13 QTL (vertical yellow arrow), a second suggestive QTL was identified on Chr 3 (diagonal black arrow). Conventions as in A. (C) Like the original Chr 13 QTL, the presence of the *B* haplotype at the Chr 3 QTL was associated with an increase in horizontal cells (red trace). Variation in *A* (green) versus *B* (red) haplotypes across Chr 3 is indicated above the map, for each of the RI strains, which have been ranked in descending order for their number of horizontal cells (shown on the right). SNP density is indicated below the map, in orange, and gene density is indicated above the haplotypes. Note that the QTL falls within a relatively gene-sparse, if also SNP-dense, portion of the chromosome. The yellow block indicates the QTL interval interrogated for candidate genes. Conventions otherwise as in A. (D) Schematic of the presence of the two haplotypes at each QTL for the 26 RI strains, plotted in descending order by horizontal cell number. The number of strains containing different combinations of the two haplotypes at these loci can be discerned, and are indicated at the bottom of E and F. (E) A two-way ANOVA revealed significant main effects of haplotype at each QTL [Chr 3: *F*_(1,22)_=19.822, *P*<0.001; Chr 13: *F*_(1,22)_=45.752, *P*<0.001], as well as a significant interaction between haplotypes [*F*_(1,22)_=9.125, *P*=0.006]. Pair-wise comparisons confirmed that for the strains having the *A* haplotype on Chr 13, the haplotype on Chr 3 does not significantly affect cell number (*P*=1.00), whereas for those strains having the *B* haplotype on Chr 13, the haplotype on Chr 3 has a significant effect on cell number (*P*<0.001). Implementing the General Linear Model without testing for an interaction reduced the adjusted R^2^ value from 0.75 to 0.66, indicating that the model with the interaction provided a better fit, accounting for 75% of the variance in horizontal cell number across the strains. (F) A two-way ANOVA performed on *Isl1* expression across the RI strains showed a significant main effect of haplotype on Chr 3 [*F*_(1,22)_=6.769, *P*=0.016] but not on Chr 13 [*F*_(1,22)_=0.303, *P*=0.59]; as with horizontal cell number, however, the interaction between haplotypes at the two chromosomes was significant [*F*_(1,22)_=14.87, *P*=0.001]. Pair-wise comparisons confirmed, as above, that for the strains having the *A* haplotype on Chr 13, the haplotype on Chr 3 does not significantly affect *Isl1* expression (*P*=1.00), whereas for those strains having the *B* haplotype on Chr 13, the haplotype on Chr 3 has a significant effect on *Isl1* expression (*P*=0.001). Finally, the model containing the interaction greatly improved the fit of the linear model, accounting for 42% of the variance in *Isl1* expression across the strains, compared with only 7% of the variance accounted for by the model without the interaction. *P<0.05.

As previously reported, the number of horizontal cells in the B6/J strain is nearly double the number present in the A/J strain, arising from differences in horizontal cell density rather than differences in retinal area (Whitney et al., 2011a). About 40% of the variance in horizontal cell number across the RI strains derived from these two parental strains (the AXB/BXA strain set) is attributed to the effect of a significant QTL on Chr 13 (Fig. 1A), indicating that variants at other loci must also modulate the number of these cells. To search for other genomic loci that modulate horizontal cell number, composite interval mapping was conducted, which maps the remaining variation in horizontal cell number that is not attributable to the QTL identified on Chr 13. Composite interval mapping revealed a second QTL on Chr 3, from approximately 119.557 to 129.579 Mb, reaching a peak logarithm of odds (LOD) score of 2.86 (Fig. 1B), where the presence of the *B* haplotype is associated with an increase in horizontal cell number (Fig. 1C). The LOD score reflects the strength of the linkage between the variation in horizontal cell number with genomic locus, as it is the logarithm of the probability that linkage exists over the probability that it does not exist. To test the significance of an LOD score, permutation testing of the strain data was performed, which enables an estimate of the probability of achieving such a score by chance. This LOD score surpassed the suggestive *P*-value threshold (0.67), but did not reach the significant *P*-value threshold (0.05), not uncommon for composite interval mapping. An alternative approach is to use the GEMMA (Genome-wide Efficient Mixed Model Association) mapping option in GeneNetwork, confirming the presence of both loci on Chrs 3 and 13 (Fig. S1). Both mapping strategies focused our attention on this newly identified additional locus on Chr 3, augmented by the evidence for its interaction with the locus on Chr 13, considered next.

When considering the 26 RI strains grouped by haplotype at both the Chr 13 and Chr 3 loci, evidence for epistasis between these QTLs was revealed (Fig. 1D). A two-way ANOVA revealed a significant interaction between haplotypes at the two loci. Post-hoc pairwise tests indicated that the strains with the *B* haplotype at both loci (Chr3/Chr13: *B/B*) had significantly greater numbers of horizontal cells than did the strains with the *A* haplotype at both loci (*A/A*) or than the strains with *A* at one locus and *B* at the other (*A/B* or *B/A*) (Fig. 1E). Crucially, for those strains with the *A* haplotype on Chr 13, the haplotype on Chr 3 did not affect cell number. By contrast, when strains had the *B* haplotype on Chr 13, the haplotype on Chr 3 significantly affected cell number. Clearly, the presence of the *B* haplotype at both loci acts synergistically to elevate horizontal cell number.

As noted above, microarray analysis previously revealed a significant negative correlation between *Isl1* expression and horizontal cell number across the RI strains (*r*=−0.53; Whitney et al., 2011a), suggesting that these two traits may be linked and that the former may also be regulated by the two QTLs. By considering *Isl1* expression as a function of haplotype status at these two controlling loci, evidence for epistasis was again revealed, as a two-way ANOVA detected a significant interaction, with the strains harboring the *B* haplotype at Chr 3 showing significantly lower *Isl1* expression, provided they also had the *B* haplotype at Chr 13 (Fig. 1F). Taken together, these results indicate that a genetic interaction exists that modulates both *Isl1* expression and horizontal cell number across the strains, directing us to interrogate this Chr 3 locus for potential candidate genes.

### Bioinformatic analysis of the Chr 3 QTL identifies *Neurog2* as a candidate gene

**Fig. 2.**
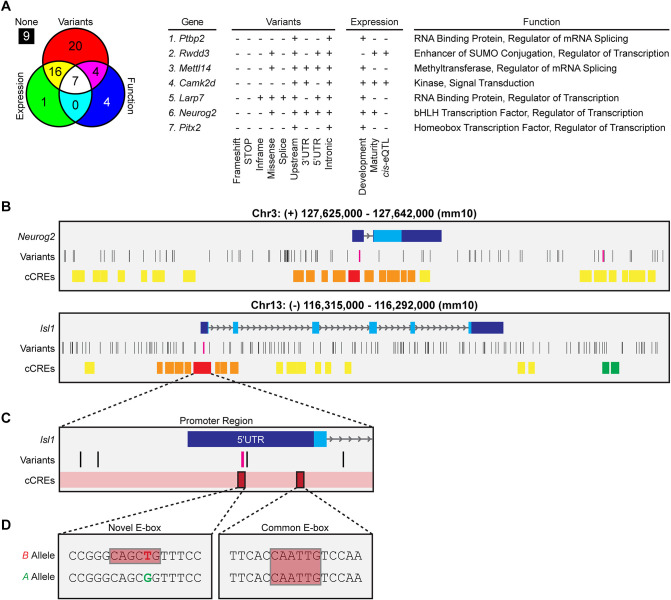
***Neurog2* is identified as a top candidate gene at the Chr 3 QTL.** (A) Bioinformatic analysis identified seven high-priority candidate genes from a total of 61 genes found at the Chr 3 QTL, based upon the presence of genetic variants discriminating the two parental genomes, their retinal expression, and their known functions. One of these candidates, *Neurog2*, was particularly noteworthy, being a bHLH transcription factor known to bind the E-box. (B) Schematics depicting *Neurog2* (top) and *Isl1* (bottom) genes (dark blue boxes indicate untranslated regions, light blue boxes indicate protein coding regions, and gray lines with arrows indicate intronic regions), including the presence of variants (e.g. SNPs, insertions/deletions, structural variants) discriminating the two parental genomes, and an indication of cCREs identified by the ENCODE Data Analysis Center (red, promoter-like signature; orange, proximal enhancer-like signature; yellow, distal enhancer-like signature; green, DNase-H3K4me3 sites). Two noteworthy candidate variants with a high probability of *cis*-modulating *Neurog2* expression are indicated (magenta variants). (C) Expanded view of the promoter region for *Isl1*, including the location of the two E-boxes in the 5′UTR and the five SNPs, one of which falls within the first E-box (magenta variant). (D) The SNP yielding the hexanucleotide sequence creating the novel E-box in *B* is shown on the left; the E-box sequence common to both genomes is indicated on the right.

The Chr 3 interval at the QTL occupies a relatively gene-sparse portion of Chr 3, a region that is itself SNP dense (Fig. 1C). This interval was interrogated for potential candidate genes likely to modulate horizontal cell number and underlie the interaction with the Chr 13 locus. A total of 61 protein-coding or microRNA-producing genes were determined to be present at the QTL; of those, we identified seven high-priority candidate genes through their known retinal expression, through the presence of potentially significant coding or regulatory variants, and through functions likely to modulate developmental processes impacting cell number (Fig. 2A). Added to that list of criteria, we paid particular attention to genes expected to alter gene expression (e.g. transcription factors, splicing factors, etc.). Of those seven candidate genes, one clearly stood out as a top candidate: *Neurog2*. This proneural gene (previously known as *Atoh4* and *Math4A*) is expressed early in retinal development as nascent neurons leave the cell cycle and proceed to differentiate (Gradwohl et al., 1996; Ma and Wang, 2006). *Neurog2* contains multiple genomic variants in regulatory regions that may alter its expression (Fig. 2B, top), and also contains one missense mutation, although the location of the altered amino acid, and the switch from a glycine to a glutamate, is not predicted to be particularly damaging (SIFT score=0.31). To explore the former more fully, we investigated sequence variants found in the 5′UTR, the 3′UTR, the single intronic region, and each of the candidate *cis*-regulatory elements (cCREs) (distal and proximal enhancers and the promoter) of *Neurog2* to determine whether they would disrupt or create transcription factor binding sites. We limited our search of binding sites to those for transcription factors known to play a role in retinal cell fate decisions and/or promote horizontal cell differentiation (e.g. ONECUT1, ONECUT2, TFAP2A, TFAP2B, LHX1, PTF1A, ATOH7, ASCL1). Two sequence variants stood out that disrupted binding sites in the *B* sequence: an SNP in the 5′UTR, near the promoter, that disrupts a TFAP2A/B binding site (GCCNNNGGC), and another SNP in a downstream distal enhancer that disrupts a bHLH transcription factor (e.g. PTF1A, ATOH7, ASCL1) binding site known as an E-box (CANNTG). Variants such as these would be expected to alter *Neurog2* expression, but by themselves do not particularly implicate *Neurog2*. Most importantly, however, *Neurog2* is itself a bHLH transcription factor, and of the many variants within *Isl1* (Fig. 2B, bottom), five occur in the putative promoter region (Fig. 2C), and one of these creates just such a novel E-box motif amongst those strains carrying the *B* haplotype at this locus (Fig. 2D), hinting at a possible mechanism underlying the interaction between the two QTLs.

### *Neurog2* CKO retinas contain fewer horizontal cells

**Fig. 3.**
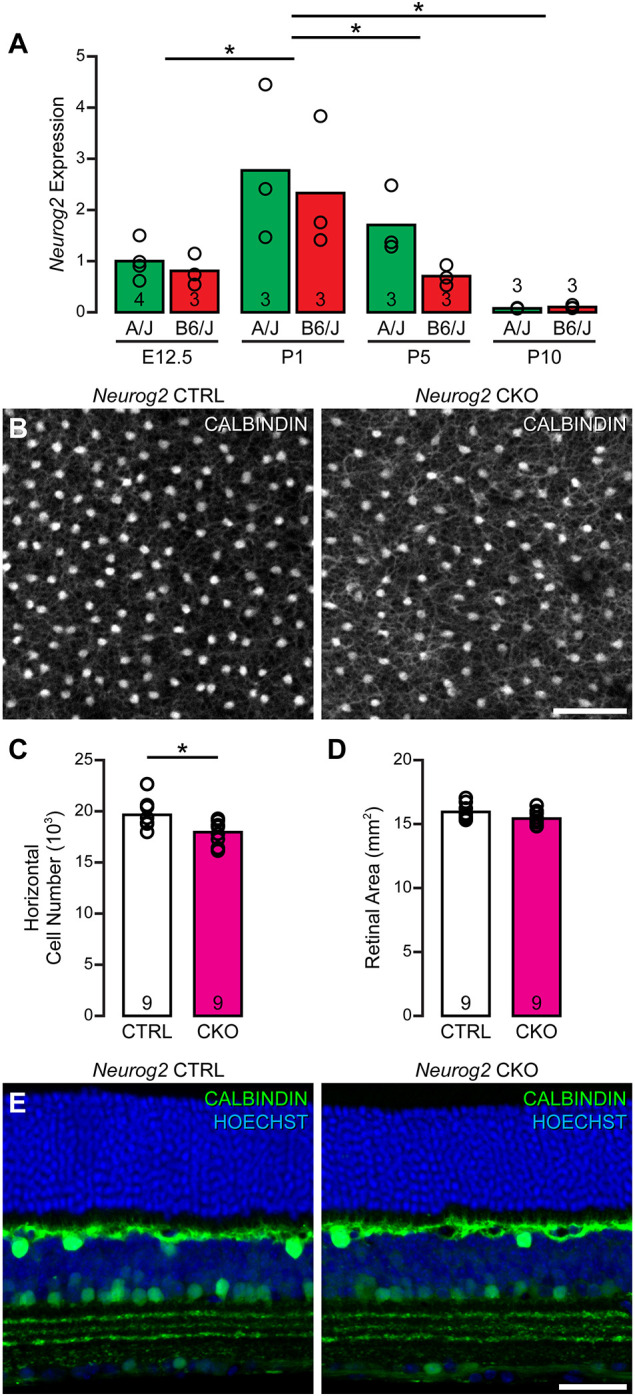
***Neurog2* CKO mice exhibit a reduction in horizontal cell number.** (A) *Neurog2* expression was assessed at four developmental ages in the two parental strains using qPCR on retinal mRNA. A trend favoring expression in A/J was present, but a two-way ANOVA confirmed only a main effect of age [*F*_(3,17)_=11.133, *P*<0.001], with expression increasing during prenatal development and declining thereafter (post-hoc Tukey tests: E12.5 versus P1, *P*=0.006; P1 versus P5, *P*=0.031; P1 versus P10, *P*<0.001). *n*=the number of samples (being pooled retinas from the same litter) per strain at each age. (B) *Neurog2* CKO retinas were compared with littermate control (CTRL) retinas for horizontal cell number in retinal wholemount preparations labeled with an antibody to calbindin. (C,D) The CKO retinas contained significantly fewer horizontal cells (one-tailed, unpaired Student's *t*-test: *P*=0.006), and exhibited a slight but non-significant change in retinal area (two-tailed, unpaired Student's *t*-test: *P*=0.079). (E) Retinal sections confirm characteristic calbindin labeling of stratified horizontal cell somata and processes, and Hoechst 33342 labeling confirms that retinal architecture is unaltered. Calibration bars=50 µm. *P<0.05.

We confirmed *Neurog2* expression during development using qPCR with retinal mRNA harvested from the two parental strains, B6/J and A/J, at embryonic day (E) 12.5, and postnatal day (P) 1, P5 and P10. A two-way ANOVA confirmed a main effect of age, with expression increasing prenatally, during the neurogenic window for horizontal cells (Hinds and Hinds, 1978; Young, 1985), climbing until P1, and declining thereafter (Fig. 3A). Although there appeared to be greater expression in A/J between E12.5 and P5, there was no significant effect of strain nor an interaction between age and strain. These results confirm the developmental regulation of *Neurog2* expression and implicate it as a potential candidate at the Chr 3 QTL interacting with *Isl1* to modulate horizontal cell number.

The presence of the B haplotype, containing the novel E-box variant in the 5′UTR of *Isl1*, is associated with a decrease in *Isl1* expression, proposed to increase the number of horizontal cells (Whitney et al., 2011a). If *Neurog2* influences *Isl1* expression via this novel E-box, then removal of *Neurog2* from the developing retina should result in a reduction in the size of the horizontal cell population. To test this hypothesis, we generated *Neurog2* CKO mice, each harboring one *Chx10-cre* allele and two copies of the floxed *Neurog2* allele, and then examined whether the number of horizontal cells was decreased in maturity compared with littermate controls (Fig. 3B). Indeed, there was a significant reduction in the number of horizontal cells, by ∼10% (Fig. 3C), confirming a role for *Neurog2* in modulating horizontal cell number, and enhancing its candidacy as a causal gene at the Chr 3 QTL. *Neurog2* CKO retinas were of comparable size to control retinas (Fig. 3D), and, consistent with other reports (Kowalchuk et al., 2018), we did not observe any defects in the retinal architecture of these *Neurog2* CKO mice. Furthermore, the population of horizontal cells was normally positioned in the outer region of the inner nuclear layer, giving rise to a dense overlapping plexus of processes as seen in wild-type retinas (Fig. 3E). Thus, it appears that although *Neurog2* promotes the acquisition of the horizontal cell fate, and in doing so modulates the final number of these cells, it is not required for horizontal cell development.

### *Neurog2* expression correlates with horizontal cell number in the RI strains carrying the novel E-box

**Fig. 4.**
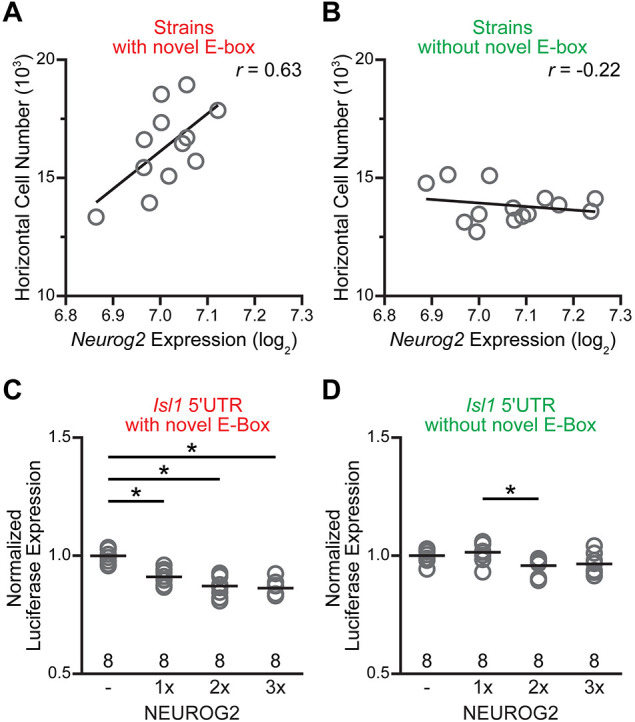
**A novel E-box in the 5′UTR of *Isl1* in B6/J mice mediates an interaction with *Neurog2*.** (A,B) Scatterplots showing the relationship between horizontal cell number and *Neurog2* expression in maturity, derived from microarray analysis of ocular mRNA from the RI strains. The RI strains have been plotted separately, according to whether they contain the *B* haplotype creating the novel E-box in the *Isl1* 5′UTR (A), or the *A* haplotype, lacking the novel E-box (B). Only in the presence of the novel E-box is there a positive correlation between horizontal cell number and *Neurog2* expression. (C,D) A luciferase expression assay examined the molecular interaction between NEUROG2 and *Isl1*. HEK293T cells were transfected with plasmids encoding the 5′UTR of *Isl1*, derived from either the B6/J genome (C) or A/J genome (D), upstream of a firefly luciferase coding sequence, and assessed through independent one-way ANOVAs [B6/J: *F*_(3,28)_=24.230, *P*<0.001; A/J: *F*_(3,28)_=4.064, *P*=0.016]. Co-transfection with a plasmid expressing the *Neurog2* coding sequence reduced luciferase output (post-hoc Tukey tests: control versus 1×, control versus 2×, control versus 3×, *P*<0.001), with increasing concentrations of these plasmids yielding a progressively stronger inhibition of luciferase, but only when using the *Isl1* 5′UTR containing the *B* sequence, creating the novel E-box. A significant difference was found between the intermediate conditions for A/J (post-hoc Tukey test: 1× versus 2×, *P*=0.032), likely driven by the slight (unexplained and non-significant) increase from the control condition at 1×. *P<0.05.

Our microarray data of adult ocular tissue (Whitney et al. 2011b) revealed meager variation in *Neurog2* expression across the RI strains, with overall levels being near the threshold for detection; this was perhaps unsurprising given the primary role of this transcription factor during development, rather than in maturity. Even so, when comparing *Neurog2* expression to horizontal number for those strains containing the *B* haplotype at Chr 13, that is, for strains in which the novel E-box is present in the 5′UTR of *Isl1*, there was a strong positive correlation (*r*=0.63) (Fig. 4A), whereas for those strains without the novel E-box, there was no relationship (*r*=−0.22) (Fig. 4B). These results suggest that the role of *Neurog2* in influencing horizontal cell number across the strains may indeed depend on the presence of the novel E-box in *Isl1*, and hint at the possibility of a direct interaction between the two genes.

### NEUROG2 directly represses *Isl1* expression through this binding motif *in vitro*

To test whether NEUROG2 reduces *Isl1* expression through the novel E-box, we created plasmids in which the 5′UTR of *Isl1* from the B6/J or A/J genome (i.e. containing the *B* or *A* variants) was cloned upstream of a Firefly luciferase coding sequence, and downstream of a constitutively active SV40 promoter. When either of these plasmids were transfected into HEK293T cells (therein allowing the simple assessment of this genetic interaction and its dependency upon the presence of the novel E-box in the absence of other factors expected to be present in retinal cells), luciferase expression increased ∼twofold over a control plasmid that contained only the promoter, indicating that the 5′UTR plays an active role in enhancing ISL1 protein levels (Fig. S2). As predicted, the level of luciferase expression significantly decreased when co-transfecting with a plasmid that expressed NEUROG2, with a progressive decline in average luciferase expression as a function of increasing levels of the transcription factor (Fig. 4C). But, crucially, this effect was only seen when using the *Isl1* 5′UTR containing the B6/J sequence, as no such trend was observed when using the *Isl1* 5′UTR containing the A/J sequence, which lacks the novel E-box (Fig. 4D). Although the second SNP present in the 5′UTR could potentially contribute an effect (Fig. 2C), it is unlikely to mediate the interaction we demonstrate here with NEUROG2, as it does not establish an E-box motif.

### Double *Isl1/Neurog2* CKO retinas contain elevated numbers of horizontal cells

**Fig. 5.**
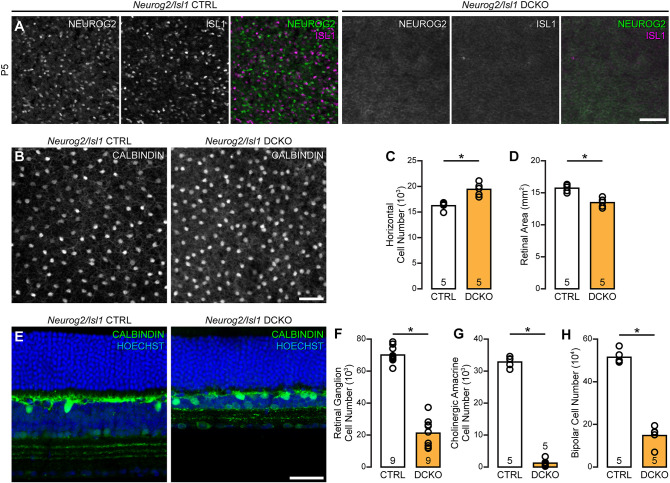
**DCKO mice reveal that *Isl1* is downstream of *Neurog2* in the regulation of retinal cell number.** (A) A *Neurog2/Isl1* DCKO mouse was generated, and confirmed via immunofluorescence to lack each respective protein, here in P5 retinal wholemounts at the level of the neuroblast layer. This age was chosen for demonstration as it is a postnatal age at which both proteins are expressed in the same layer; *cre*-mediated recombination in the *Chx10-cre* mouse has been observed in retinal progenitor cells as early as E10.5 (Rowan and Cepko, 2004), however, and therefore gene excision is expected to have occurred prior to horizontal cell genesis. (B) DCKO retinas were similarly assessed for horizontal cell numbers relative to littermate controls in retinal wholemounts. (C,D) DCKO retinas showed a significant increase in horizontal cell numbers (two-tailed, unpaired Student's *t*-tests: *P*=0.001), as found in the *Isl1* CKO retina (Whitney et al., 2011a), despite a decrease in overall retinal area (*P*=0.001). Note that the control retinas here differ from those shown in Fig. 3 in the size of their horizontal cell populations because of the distinct genetic backgrounds associated with the mice contributing the floxed alleles. (E) Stratification and differentiation of horizontal cells in the DCKO retina appeared unaltered, despite the conspicuous reduction of cells in the inner retina. (F-H) DCKO retinas exhibited large and significant declines in the populations of *Isl1*-expressing neurons, including retinal ganglion cells (two-tailed, unpaired Student's *t*-tests: *P*<0.001), cholinergic amacrine cells (*P*<0.001) and bipolar cells (*P*<0.001), as reported by others in the *Isl1* CKO retina (Elshatory et al., 2007). *n*=the number of mice examined in each group. CTRL, control. Scale bars: 50 µm. *P<0.05.

Given the synergistic interaction between the QTLs on Chrs 3 and 13, the opposing effects upon horizontal cell number seen in each respective single CKO mouse, and the ability of NEUROG2 to directly repress *Isl1* in the presence of the novel E-box *in vitro*, these two genes are likely involved in the same developmental process to modulate acquisition of the horizontal cell fate *in vivo*, with *Neurog2* working upstream of *Isl1* in an antagonistic manner. Mice in which both *Neurog2* and *Isl1* are eliminated, therefore, should simply mimic the *Isl1* CKO, leading to an increase in horizontal cell number (Whitney et al., 2011a). DCKO mice were generated (Fig. 5A), and, indeed, found to contain a significant increase in horizontal cell numbers (Fig. 5B,C), as in the *Isl1* CKO mouse (Whitney et al., 2011a). These cells appeared otherwise normal in their positioning and morphology (Fig. 5E), despite a conspicuous (13%) reduction in retinal area (Fig. 5D) and major reductions in other inner retinal neuronal populations, including the retinal ganglion cells (RGCs), cholinergic (ChAT) amacrine cells, and bipolar cells (Fig. 5F-H); these populations all contain *Isl1*-expressing cells in maturity, and these reductions in cell number mirror the effects observed in the *Isl1* CKO mouse (Elshatory et al., 2007). Taken together, the results of this study directly demonstrate the genetic interaction by which *Neurog2* reduces *Isl1* expression and its dependency upon a sequence variant in the 5′UTR, which we propose to underlie the epistatic interaction between the QTLs controlling horizontal cell number.

## DISCUSSION

### QTL mapping reveals an epistatic interaction controlling horizontal cell number

Retinal cell number, for various cell types, shows marked variation between different strains of mice (Keeley et al., 2014; Williams et al., 1998a,b), and that variation is typically graded across strains, indicative of the polygenic nature of such traits. QTL mapping frequently reveals multiple loci acting in concert to determine final cell number, and dissection of those loci identify prospective genes contributing to those QTL effects. Multiple variant genes, in some instances contained within the same locus, modulate cell number additively (Whitney et al., 2009), occasionally exhibiting countervailing effects to render the parental strains hardly different (Kautzman et al., 2018a,b; Keeley et al., 2017). AII amacrine cells, for instance, hardly differ between the two parental strains, B6/J and A/J, yet their RI strains vary by nearly 30,000 cells. There, the graded variation across the strains shows a few pronounced step-like changes, implying a couple of variant genes having outsized effects relative to other participants, and QTL analysis reveals two large-effect loci on Chrs 9 and 19. The loci are equivalent in their effects, together accounting for ∼75% of the variation across the strain set, where the *A* haplotype at each locus elevates AII amacrine cell number (Kulesh et al., 2019). By parsing those strains according to haplotype at the two genomic loci on Chrs 9 and 19, a clear additive relationship is revealed, with *A/A* strains containing numbers of cells that approximate the additive effects exhibited by the presence of the *A* versus *B* haplotype at each locus independently (i.e. in the *A/B* and *B/A* strains) (Kulesh et al., 2019). Here, we have identified a secondary QTL modulating horizontal cell number, one that accounts for a smaller effect when mapped alone (Fig. 1C), but which has a synergistic effect when in the presence of one, but not the other, haplotype at the primary QTL (Fig. 1D,E). This synergy in affecting the number of horizontal cells is complemented by a comparable synergy in the repressive effect upon *Isl1* expression when in the presence of the *B* haplotype at both loci (Fig. 1F). Their combined contributions, of course, would not be expected to account for the entirety of the variation in horizontal call number, given the presence of other lesser contributing genomic loci. The effects upon cell number in the CKO retinas should likewise not be expected to account for that entire variation for the same reason, in addition to the fact that the Cre-mediated recombination is not complete when using this *Chx10-cre* allele (Rowan and Cepko, 2004).

### The regulation of *Isl1* expression by NEUROG2 shown *in vitro* is proposed to mediate the epistatic interaction between the two QTLs

The SNP in the *Isl1* 5′UTR that creates a novel E-box had been proposed previously to mediate the repression of *Isl1*, and in doing so, elevating horizontal cell number (Whitney et al., 2011a). Here, we provide evidence that this repression is under the control of NEUROG2. First, we confirm the developmental upregulation of *Neurog2* expression during the prenatal period, when horizontal cells are being generated, and demonstrate that loss of this bHLH transcription factor yields a decline in the number of horizontal cells, opposite in action to the loss of *Isl1* upon horizontal cell number. Second, we show that *Neurog2* expression across the RI strains correlates with horizontal cell number, but only in the presence of the novel E-box produced in strains carrying the *B* sequence at the Chr 13 locus, and we provide direct evidence for this interaction between NEUROG2 and the B6/J *Isl1* 5′UTR containing the novel E-box using an *in vitro* luciferase expression assay. Finally, we show that the decremental effect of losing *Neurog2* upon horizontal cell number is abrogated when *Isl1* is also eliminated, in the DCKO retina, yielding an increase in their number, as found in the *Isl1* CKO retina (Whitney et al., 2011a), confirming that *Neurog2* works upstream to repress *Isl1* expression *in vivo*. Taken together, these results suggest that in the early developing retina, NEUROG2 works to repress *Isl1* and in turn promote the acquisition of the horizontal cell fate.

The presence of the *B* sequence of the *Isl1* 5′UTR creating this E-box either completely enables or at least enhances this interaction, which should contribute to the modulation of *Isl1* expression across the AXB/BXA RI strains. There are several other E-boxes throughout the cCREs of *Isl1*, including a second E-box present in the 5′UTR of Isl1 (Fig. 2C,D), common to both genomes. Despite this common E-box being present, the increasing concentrations of NEUROG2 used in our luciferase assay in the presence of the *A* sequence failed to produce a graded decrement in luciferase output, suggesting a difference in the sensitivity of the two E-boxes. In support of this, the common E-box, despite having the universal CANNTG motif, has atypical nucleotides in the variable region; although the two middle nucleotides within this hexanucleotide motif can be any nucleotides, unique bHLH factors can exhibit differential affinity to some combination over others, including NEUROG2 (Aydin et al., 2019), and the novel E-box has the canonical E-box sequence CAGCTG. To this point, transcription factor binding site prediction databases identify it with high confidence, but, conversely, fail to identify the common E-box sequence CAATTG altogether. Although we cannot rule out the possibility that the common E-box, or any of the other predicted E-boxes outside the 5′UTR, plays a role in the regulation of *Isl1* by NEUROG2 *in vivo*, the location and sequence of the novel E-box should render it a highly significant contributor to overall *Isl1* expression.

We have not yet found such a single causal variant within *Neurog2* at the QTL on Chr 3. Given the lack of potential candidates that might alter NEUROG2 functionality, we suspect that the causal variant (or variants) at the Chr 3 QTL is more likely to modulate the expression of *Neurog2*, and, in doing so, alter the overall repression of *Isl1* via the novel E-box. This would be consistent with the positive correlation we observed between *Neurog2* expression and horizontal number for those strains containing the E-box, and the greater repression of *Isl1* with increasing concentrations of NEUROG2 in our luciferase assay. We found 24 variants that discriminate between the A/J and B6/J genomes that reside in cCREs as predicted by the GENCODE project (Fig. 2B), noting two SNPs that may affect the expression of *Neurog2* by altering binding sites for transcription factors known to modulate horizontal cell fate.

### The role of NEUROG2 in the control of horizontal cell number

NEUROG2 is a pro-neural factor and one of the bHLH transcription factors that is expressed in transitional retinal progenitor cells, along with other bHLH factors, such as ASCL1, ATOH7, NEUROD1 and OLIG2 (Brzezinski et al., 2011; Feng et al., 2010; Hafler et al., 2012; Morrow et al., 1999). As these transitional progenitor cells leave the cell cycle, they exhibit heterogeneity in the expression patterns of such factors (Brzezinski et al., 2011; Trimarchi et al., 2008), interpreted as indicating different competence states. Interestingly, progenitors that express many of these factors can give rise to almost all cell classes in the retina, while simultaneously promoting specific cell fates over others. For example, *Atoh7* (previously known as *Math5*) is necessary for RGC genesis (Brown et al., 2001; Wang et al., 2001), committing cells to this fate by directly activating RGC gene *Pou4f2* (*Brn3b*) and *Isl1* (Wu et al., 2021, 2015), and, furthermore, expression is correlated with RGC genesis (Prasov et al., 2012; Zhang et al., 2018; but see Prasov and Glaser, 2012). Yet progenitors that express this bHLH factor are not guaranteed to become RGCs, as they can also give rise to horizontal cells, photoreceptors and amacrine cells (Feng et al., 2010). *Neurog2* could be acting similarly to promote the horizontal cell fate, even as neurogenic cells that express this factor give rise to all major neuronal classes in the retina (Brzezinski et al., 2011).

Previous studies that knocked out combinations of bHLH factors in the developing retina noted that horizontal cell number was reduced only in combinations in which *Neurog2* was removed (Akagi et al., 2004), forming the first clue that this gene may be important for the determination of the horizontal cell fate. An initial study of single knockout mice showed an increase in RGCs, indicating that *Neurog2* normally acts to suppresses RGC fate, but the authors reported no effect on the numbers of the other early-born retinal neurons, including cones, amacrine cells and horizontal cells (Hufnagel et al., 2010). However, all PROX1-positive cells in the inner nuclear layer were quantified to assess horizontal cell and amacrine cell number together; horizontal cells were not assessed independently (Hufnagel et al., 2010). Subsequent studies looked at the composition of later-generated cell types and observed an increase in bipolar cells at the expense of rod photoreceptors, while confirming a lack of change in the numbers of amacrine cells and cone photoreceptors (Kowalchuk et al., 2018). These researchers, however, did not quantify the number of horizontal cells in these knockout mice either. Thus, to our knowledge, the present results are the first to demonstrate a reduction in the number of horizontal cells in the absence of *Neurog2* alone.

The effect of *Neurog2* on horizontal cell number is modest, and the transcription factor is clearly not required for proper horizontal cell development. Rather, it appears that *Neurog2* expression in transitional retinal progenitor cells serves to compete with some bHLH factors, such as *Atoh7*, while likely coordinating with others, to ultimately define the competence of the cell as it proceeds down a differentiation pathway. *Neurog2* may tip the balance in favor of a horizontal cell fate, perhaps through the activation of pro-horizontal cell genes (e.g. *Prox1*, *Lhx1*) and/or repressing pro-RGC genes (e.g. *Isl1*). It is interesting to note that *Neurog2* seems to suppress rod bipolar cell fate, another cell type that requires *Isl1* for proper development, while promoting rod photoreceptor genesis instead (Kowalchuk et al., 2018), suggesting that the same fate-determination dynamic might play out in later-born retinal neurons as well. By utilizing the genetic and phenotypic variation present across different mouse strains, we can continue to unravel the dynamics of gene interactions that shape the size and composition of neuronal populations in the retina.

## MATERIALS AND METHODS

### Mice

Profiles of each of the transgenic mice and inbred strains used in the current study are given in Table S1. CKO and DCKO mice were produced by breeding mice containing floxed alleles of *Isl1* and/or *Neurog2* with *Chx10-cre* mice. The single transgenic mice were not backcrossed, so the resultant offspring displayed a high amount of genetic heterogeneity; therefore, littermate control mice were used in each separate CKO analysis, being mice lacking either the respective floxed allele(s) or the *Chx10-cre* allele. The original parental strains, A/J and B6/J, were used to profile *Neurog2* expression during embryonic and postnatal development. Recombinant inbred strains from the AXB/BXA strain set were previously analyzed for horizontal number and used for the initial QTL mapping; details on these mice (all females, 1-3 months of age), the quantification procedure, and the original mapping are described elsewhere (Whitney et al., 2011a). All procedures using animals were approved by the Institutional Animal Care and Use Committee at the University of California, Santa Barbara, and were in accordance with the NIH Guide for the Care and Use of Laboratory Animals.

### Immunofluorescence

Mice between 6 and 8 weeks of age were given an intraperitoneal injection of a lethal dose of sodium pentobarbital (120 mg/kg). Once deeply anesthetized, they were perfused through the heart with 2-3 ml of 0.9% saline followed by ∼75 ml of 4% paraformaldehyde in 0.1 M sodium phosphate buffer (PB; pH 7.2-7.4). Both eyes were then dissected from the orbits and immersed in the same fixative for 15 min. Alternatively, *Neurog2/Isl1* control and DCKO mice of the same litter at postnatal day (P) 5 were euthanized, and their eyes were then swiftly dissected and immersed in the same fixative for a total of 30 min. Retinal wholemounts or 200 µm thick radial sections cut on a PELCO easiSlicer were prepared for immunofluorescence, all as recently described (Kulesh et al., 2022). A list of the primary and secondary antibodies used is provided in Table S2. In short, these were used to confirm the loss of ISL1 and/or NEUROG2 in CKO retinas, or to identify classes of retinal neurons for quantification, including horizontal cells, bipolar cells, ChAT^+^ amacrine cells and retinal ganglion cells, as indicated in Table S2. When staining retinal sections, Hoechst 33342 (1:1000; H3570, Thermo Fisher Scientific) was added to the secondary antibodies to identify the nuclear layers.

### Quantification of retinal cell types

Immunolabeled retinal wholemounts were imaged using an Olympus FluoView FV1000 laser-scanning confocal microscope. Eight fields were sampled for each labeled population from a single retina of each mouse, at central and peripheral eccentricities in each quadrant, using a ×40 objective. The eight sampled images from every CKO and littermate control retina were given coded identifiers and randomly interleaved by one investigator and then all coded fields were counted by a second investigator, being thereby blind to the identity of each sampled field and precluding batch effects that might be contaminated by criterion drift. For each type of cell identified, an average density was calculated for each retina and multiplied by retinal area to estimate the total number of cells. The size of each sampled field for each of the cell types is indicated in Table S2.

### QTL mapping

Simple and composite interval mapping were conducted on the RI strain data for horizontal cell number (phenotype AXB_10132) in the AXB/BXA phenotypes database of GeneNetwork (version 1; gn1.genenetwork.org), and for *Isl1* and *Neurog2* expression in the AXB/BXA ocular mRNA expression database (GN210; probes ILMN_2727472 and ILMN_1213024, respectively; Whitney et al., 2011b), using the mapping module in GeneNetwork. Because the genome of each RI strain is a unique mix of the parental haplotypes following the nearly random recombination events occurring over the course of inbreeding each RI strain, QTL mapping can estimate the covariance between the trait of interest and the presence of the *A* or *B* haplotype across the genome. GeneNetwork implements standard methods for simple and composite interval mapping to identify controlling genomic loci, estimating the genome-wide *P*-value of a false-positive error by randomly permuting the strain data 2000 times to determine suggestive (*P*<0.67) and significant (*P*<0.05) thresholds for the LOD score. The LOD score is an index of the strength of the linkage between the variation in our trait of interest (here, either horizontal cell number or gene expression) and genomic locus. Composite interval mapping was performed in GeneNetwork to control for the variation in horizontal cell number observed at the primary QTL on Chr 13 using the genetic background SNP marker rs4230072 (112.738158 Mb; genome assembly mm10), as this marker had the highest LOD score at the Chr 13 QTL. GEMMA (Zhou and Stephens, 2012) was performed using GeneNetwork (version 2; www.genenetwork.org) as an alternative mapping strategy to confirm the results of the simple and composite interval maps. GEMMA runs linear mixed models to test specifically for the effect of relatedness between strains on the association between genotype and the quantified trait (e.g. horizontal cell number).

### Candidate gene analysis

The proximal and distal boundaries of the Chr 3 QTL were defined as the locations on either side of the peak where the LOD score was equal to 1.36 (a 1.5 LOD decrease from the peak LOD), and a list of each gene present at the interval was generated using the mm10 genome assembly. We then conducted a bioinformatic assessment to identify potential candidate causal genes. All genes within the interval lacking genomic variants discriminating the parental genomes were eliminated from further consideration, whereas remaining genes that had been shown to be expressed in retina, particularly during development, and associated with functions likely to modulate the size of a cellular population, were given further scrutiny. We identified every candidate genomic variant present in each of these higher priority candidate genes, including all SNPs, insertions/deletions, and structural variants discriminating the parental strain genomes, using Release-1505 (GRCm38) of the Mouse Genomes Project from the Wellcome Sanger Institute (Doran et al., 2016; Keane et al., 2011). Variants were subsequently classified as either potential regulatory or functional variants based on their location within each gene. A detailed analysis of the genomic structure of both *Neurog2* and *Isl1* (gene structures identified using the NCBI RefSeq database) was performed to rank potential causal variants. The location of regulatory variants was cross-referenced with cCREs identified from the ENCODE project (www.encodeproject.org) and potential transcription factor binding motifs predicted from the JASPAR 2022 database (jaspar.genereg.net), both visualized using publicly available custom tracks in the UCSC genome browser. Additionally, the potential consequence of missense variants was assessed using SIFT (sift.bii.a-star.edu.sg).

### Quantitative (q)PCR

Fresh postnatal retinas were collected from P1, P5 and P10 B6/J and A/J mice and placed into RNAlater (Bio-Rad), taking precautions to avoid RNase contamination; mRNA was subsequently purified using an RNeasy Mini Kit (74104, QIAGEN). Additionally, mRNA was collected from embryonic day (E) 12.5 A/J and B6/J mice as described previously (Whitney et al., 2011a). In all cases, samples comprised pooled retinas from all of the mice in the same litter (minimum of three mice), and single-stranded cDNA was produced from each sample using an iScript cDNA synthesis kit with oligo(DT) and random primers (1708890, Bio-Rad). Each sample was run on the same plate in triplicate using a CFX96 qPCR thermal cycler (Bio-Rad) to detect the abundance of *Neurog2*; separate plates were run with primers to detect glyceraldehyde 3-phosphate dehydrogenase (*Gapdh*) and β-2 microglobulin (*B2m*) to assess the abundance of these internal control (e.g. housekeeping) genes. For each sample, the Ct values were adjusted based on the average efficiency of the PCR reaction (determined by performing linear regressions of each reaction during the geometric phase), and the median adjusted value across the three technical replicates for *Neurog2* was normalized by the average of the median for the two internal control genes. Finally, all expression values were normalized to the average expression at E12.5 in the A/J strain for comparison across strain and age. Details for the primers used for qPCR are listed in Table S3.

### Luciferase assay

Plasmids were created to test the regulatory effect of *Neurog2* upon the *Isl1* 5′UTR containing the *A* versus *B* variants in an *in vitro* luciferase expression assay. The *Neurog2* coding sequence was amplified from cDNA sample generated from the brain of a 1-day-old B6/J mouse, and then cloned into the pTARGET Mammalian Expression Vector (A1410, Promega), in which the expression of *Neurog2* is driven by the CMV promoter. The entire 5′UTR of the *Isl1* gene was amplified from genomic DNA samples from B6/J and A/J mice and cloned into a pGL3-promoter plasmid (E1761, Promega), upstream of the firefly luciferase coding sequence and downstream of the SV40 promoter. Empty pTARGET plasmids were used to normalize transfection conditions, such that every well received the same total molar equivalent of plasmids; additionally, a pRL-TK plasmid (E2241, Promega), in which the TK promoter drives the expression of *Renilla* luciferase, was added to every well as a transfection control.

HEK293T cells (originally obtained from ATCC and then frozen at passage 6; RRID:CVCL_0063) were seeded into 24-well plates at a density of 50,000 cells per well and grown in DMEM+GlutaMAX (10566016, Thermo Fisher Scientific) fortified with 10% fetal bovine serum (A3160401, Thermo Fisher Scientific) and a cocktail of penicillin and streptomycin (100 U/ml and 100 μg/ml, respectively; 15140122, Thermo Fisher Scientific) to prevent contamination. Twenty-four hours after seeding, cells were transfected with a combination of plasmids using TurboFect (R0531, Thermo Fisher Scientific); the next day, protein extracts from each well were collected using Glo Lysis Buffer (E2661, Promega) and split into four wells of a white-bottomed 96-well plate. Steady-Glo reagent (E2510, Promega) was added to two wells to assess firefly luciferase activity, and *Renilla*-Glo reagent (E2710, Promega) was added to the other two wells to determine *Renilla* luciferase activity. Luminosity readings were taken using a Perkin Elmer Wallac 1420 microplate reader and normalized as follows: first, technical replicates were averaged, then the ratio of firefly luciferase to *Renilla* luciferase was calculated for each culture well to account for differences in transfection efficiency. Finally, to compensate for variation across different plates, which were run at separate times with cells at different passages, the readings for each culture well were normalized to the average reading of the control condition for each plate. In total, three plates were analyzed, using sequential passages of cells.

### Statistics

One-way ANOVA was used to examine for differences in horizontal cell number, and in *Isl1* expression from microarray analysis, between the different RI strains sorted by haplotype at Chrs 3 and 13, whereas post-hoc Tukey tests were used to assess significant group differences. Two-way ANOVA and post-hoc Tukey tests were used to assess *Neurog2* expression from qPCR analysis between the parental B6/J and A/J strains at different developmental ages. Two-way ANOVAs were performed using the General Linear Model in SPSS (Version 26; IBM) to test for possible interactions of haplotype at the QTLs Chr 3 and Chr 13 on horizontal cell number as well as *Isl1* expression from the microarray analysis. Significant interactions were observed in both cases; thus, post-hoc pairwise tests using the Bonferroni adjustment for multiple tests were performed using the stats package in R (rdocumentation.org/packages/stats/) to detect significant differences between groups. For horizontal cell number, the average age for each recombinant strain was also added as a covariate; no significant effect of age was detected (*P*=0.64), and thus age was removed as a covariate factor in subsequent analyses. Likewise, the age of mice used for the microarray analysis did not significantly contribute to the linear model fit to *Isl1* expression across the strains (*P*=0.83). To test the contribution of the interaction between QTL on the fit of the linear model, two models were generated, with and without the interaction factor (Chr 3 * Chr 13), and the differences in fit (adjusted R^2^) between the models were compared. To confirm the robustness of the effects, ANOVAs were additionally conducted following both log_2_ and quantile transformation of the horizontal cell number data using the transform module of GeneNetwork (version 2), finding comparable statistical outcomes. None of the strains was confirmed to constitute outliers for horizontal cell number, and although AXB5 was identified as an outlier among strains for *Isl1* expression, removal of this strain from the analysis did not alter the outcome of the statistical analysis.

Two-way ANOVA was used to assess *Neurog2* expression (from the qPCR analysis) between the parental B6/J and A/J strains at different developmental ages. As no significant interaction between strain and age was detected, significant differences across ages were detected using post-hoc Tukey tests. Student's one-tailed *t*-tests were used to assess for significant differences in horizontal cell number between CKO mice and their respective littermate controls, as per prediction, and two-tailed *t*-tests were used to detect significant differences in other cell types. One-way ANOVAs were used to assess differences in luciferase expression across conditions *in vitro*, followed by post-hoc Tukey tests. An alpha threshold of 0.05 was used for determining statistical significance in all cases, indicated by an asterisk; individual *P*-values are indicated in the figure legends.

## Supplementary Material

10.1242/develop.201315_sup1 Supplementary information
