# Supplementary material for: Neurog2 regulates Isl1 to modulate horizontal cell number
Source: Development. 2023 Jan 5;150(1):dev201315. doi: 10.1242/dev.201315 (PMC10108602; doi:10.1242/dev.201315)
Supplement: Supplementary information [file develop-150-201315-s1.pdf]

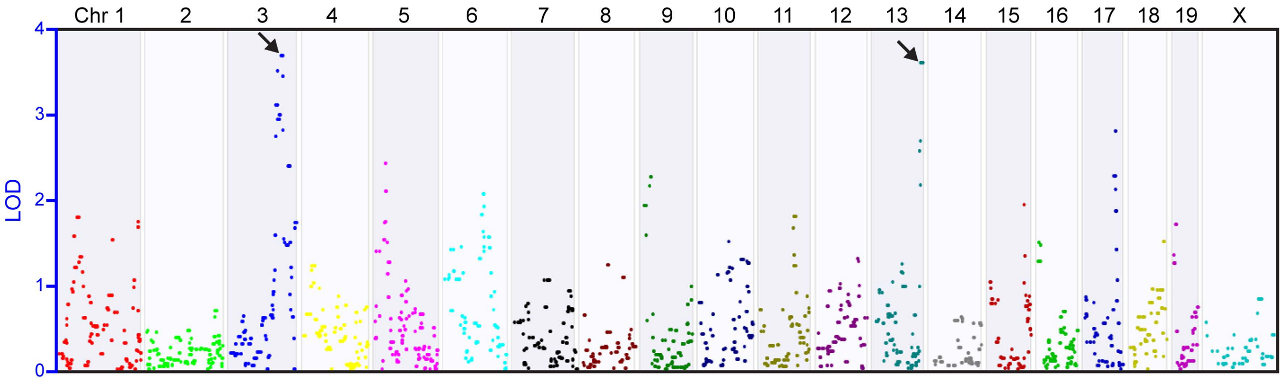

Fig. S1. GEMMA mapping reveals QTL on Chromosomes 3 and 13. The Genome-wide Efficient Mixed Model Association (GEMMA) mapping module in GeneNetwork uncovers the same two QTL on Chrs 3 and 13 as identified in Fig. 1A and B.

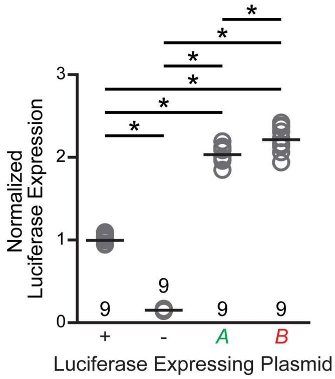

Fig. S2. Addition of the *Isl1* 5′UTR enhances luciferase expression. In HEK293T cells, a luciferase expressing plasmid with a consitutively active SV40 promoter (+) significantly increased luciferase expression over a promoterless plasmid (-). Addition of the *Isl1* 5′UTR upstream of the luciferase coding sequence, doubled the luciferase output, whether using the sequence derived from A/J (*A*) or B6/J (*B*) strain. A one-way ANOVA revealed significant differences between conditions ($F_{(3,32)}$=826.919, P<0.001), with post-hoc Tukey tests indicating significant differences between each pairwise comparison (all comparisons, P<0.001, except A vs B, P=0.003). *=significant difference detected, P<0.05.

Development • Supplementary information

Table S1. Mice used in this study

Name	Transgene	Function	Citation	RRID
C57BL/6J	None	Inbred strain; one of the parental strains of the AXB/BXA strain-set	N/A	RRID:IMSR_JAX:000664
A/J	None	Inbred strain; one of the parental strains of the AXB/BXA strain-set	N/A	RRID:IMSR_JAX:000646
Chx10-cre	Tg(Chx10-EGFP/cre,-ALPP)2Clc	Expresses cre recombinase in early retinal progenitors (~E10.5)	Rowan and Cepko, 2004	RRID:IMSR_JAX:00510 5
Neurog2-flox	Neurog2tm5(Neurog2)Fgu	Entire coding sequence of *Neurog2* flanked by *loxP* sites to create conditional knockout mice	Hand et al., 2005	RRID:MGI:3703640
Isl1-flox	Isl1tm2Sev	Exon 4 of *Isl1* flanked by *loxP* sites to create conditional knockout mice	Sun et al., 2008	RRID:IMSR_JAX:028501

References

Rowan, S. and Cepko, C. L. (2004). Genetic analysis of the homeodomain transcription factor Chx10 in the retina using a novel multifunctional BAC transgenic mouse reporter. *Dev. Biol.* **271**, 388-402. doi:10.1016/j.ydbio.2004.03.039

Hand, R., Bortone, D., Mattar, P., Nguyen, L., Heng, J. I., Guerrier, S., Boutt, E., Peters, E., Barnes, A. P., Parras, C. (2005). Phosphorylation of Neurogenin2 specifies the migration properties and the dendritic morphology of pyramidal neurons in the neocortex. *Neuron* **48**, 45-62. doi:10.1016/j.neuron.2005.08.032

Sun, Y., Dykes, I. M., Liang, X., Eng, S. R., Evans, S.M. and Turner, E. E. (2008). A central role for Islet1 in sensory neuron development linking sensory and spinal gene regulatory programs. *Nat. Neurosci.* **11**, 1283-1293. doi:10.1038/nn.2209

Table S2. Antibodies used in this study

Target	Host	Dilution	Cell Type Counted	Field Size	Catalog #	RRID
NEUROG2	Rabbit	1:1000	N/A	N/A	GTX129258 (GeneTex)	RRID:AB_2885944
ISL1	Mouse	1:500	N/A	N/A	39.4D5 (DSHB Uni of Iowa)	RRID:AB_2314683
CALBINDIN	Rabbit	1:2500	Horizontal Cells	0.101 mm^2	PC253L (Millipore)	RRID:AB_213554
CHAT	Goat	1:250	Cholinergic Amacrine Cells	0.101 mm^2	AB144P (Millipore)	RRID:AB_2079751
RBPMS	Guinea Pig	1:1000	Retinal Ganglion Cells	0.011 mm^2	1832-RBPMS (PhosphoSolutions)	RRID:AB_2492226
OTX2	Goat	1:500	Bipolar Cells	0.003 mm^2	AF1979 (R&D Systems)	RRID:AB_2157172
Mouse IgG	Donkey	1:200	N/A	N/A	A-31570 (Invitrogen)	RRID:AB_2536180
Rabbit IgG	Donkey	1:200	N/A	N/A	A-21206 (Invitrogen)	RRID:AB_2535792
Goat IgG	Donkey	1:200	N/A	N/A	A-21447 (Invitrogen)	RRID:AB_2535864
Guinea Pig IgG	Donkey	1:200	N/A	N/A	706-585-148 (Jackson ImmunoResearch)	RRID:AB_2340474

Table S3. Primers used for qPCR

Target	Primer	Sequence (5′-3′)	Amplicon Size	Annealing Temp	Average Efficiency
Neurog2	Forward	CCCTGTGATCTTTTCACCTTCG	188 bp	61.5°C	1.98
	Reverse	AGGGAAAGTTTGGTTTGACAGG			
Gapdh	Forward	AATGTGTCCGTCGTGGATCTGA	117 bp	63.0°C	1.94
	Reverse	AGTGTAGCCCAAGATGCCCTTC			
B2m	Forward	GGAGAATGGGAAGCCGAACATAC	143 bp	63.0°C	1.98
	Reverse	AGAAAGACCAGTCCTTGCTGAAG			

Development • Supplementary information
