# Supplementary material for: Neurog2 regulates Isl1 to modulate horizontal cell number
Source: Development. 2023 Jan 5;150(1):dev201315. doi: 10.1242/dev.201315 (PMC10108602; doi:10.1242/dev.201315)
Supplement: Supplementary file 1 [file develop-150-201315_review_history.pdf]

Neurog2 regulates *Isl1* to modulate horizontal cell number

Patrick W. Keeley, Pooja S. Patel, Matthew S. Ryu and Benjamin E. Reese
DOI: 10.1242/dev.201315

Editor: Francois Guillemot

Review timeline
Original submission: 22 September 2022
Editorial decision: 21 October 2022
First revision received: 18 November 2022
Accepted: 6 December 2022

Original submission

First decision letter

MS ID#: DEVELOP/2022/201315

MS TITLE: Neurog2 Regulates Isl1 Expression to Modulate Horizontal Cell Number

AUTHORS: Patrick W Keeley, Pooja S Patel, Matthew S Ryu, and Benjamin E Reese

I have now received the reports of three referees on your manuscript and I have reached a decision. The referees' comments are appended below, or you can access them online: please go to BenchPress and click on the 'Manuscripts with Decisions' queue in the Author Area.

As you will see, all the referees express great interest in your work, but they also have significant criticisms and recommend a substantial revision of your manuscript before we can consider publication. In particular, they request that you strengthen, both statistically and in the text, the argument that Neurog2 and Isl1 interact. If you are able to revise the manuscript along the lines suggested, which may involve further experiments, I will be happy to receive a revised version of the manuscript. Your revised paper will be re-reviewed by one or more of the original referees, and acceptance of your manuscript will depend on your addressing satisfactorily the reviewers' major concerns. Please also note that Development will normally permit only one round of major revision. If it would be helpful, you are welcome to contact us to discuss your revision in greater detail. Please send us a point-by-point response indicating your plans for addressing the referee's comments, and we will look over this and provide further guidance.

Please attend to all of the reviewers' comments and ensure that you clearly highlight all changes made in the revised manuscript. Please avoid using 'Tracked changes' in Word files as these are lost in PDF conversion. I should be grateful if you would also provide a point-by-point response detailing how you have dealt with the points raised by the reviewers in the 'Response to Reviewers' box. If you do not agree with any of their criticisms or suggestions please explain clearly why this is so.

Reviewer 1

Advance summary and potential significance to field

In this paper, the authors investigate the genetic underpinning of the variations of retinal horizontal cell numbers Ammon different mouse strains.
Extending from their previous finding identifying that SNPs in the Isl1 gene locus are significantly associated with horizontal cell number variations, in the current study the authors identify the

© 2023. Published by The Company of Biologists under the terms of the Creative Commons Attribution License (https://creativecommons.org/licenses/by/4.0/).

locus of the Neurogenin2 gene is also associated with this trait. Further, the authors identify an epistatic relationship between Neurogenin2 and Isl1; Neurogenin2 represses Isl1 to overcome the negative effect of Isl1 on horizontal cell numbers via an E box present in one haplotype. They show supportive evidence of the epigenetic relationship by analyzing single and double knockout mice and reporter assays.
This is an interesting study overall, addressing an important issue regarding how cell numbers are controlled in the retina. The data are clear and convincing and the paper should be of interest to readers of Development.

Comments for the author

Some of the critiques I have are detailed below:
1. It is clear that Neurogenin2 and Isl1 are not responsible for all the differences between the two stains studies in horizontal cell numbers, as the differences revealed by the knockouts are much smaller. Other variations may be at play too. The authors should make this clear in the discussion.
2. The significance of the E box in the Isl1 5' UTR in the epistasis between Neurogenin2 and Isl1 is not fully established, although results from the reporter assay are supportive. It would be more convincing if the authors could show that in the haplotype not carrying the E box, knockout of Neurogenin2 makes no difference as in the case when Isl1 is knocked out
3. How the variations in the Neurogenin2 locus affect its function is vague. More discussion is desired.

Reviewer 2

Advance summary and potential significance to field

This paper describes the mapping of a locus that influences the number of retinal horizontal cells. The authors have previously identified a gene, Is1, on chromosome 1, and now map a second locus on chromosome 13 that contains Neurog2. They present evidence from knockouts and luciferase reporter assays to demonstrate the involvement of this gene also in cell number variation.

Comments for the author

First, despite claims that they have identified the gene underlying the chr3 QTL (e.g. Page 8 line 166 "confirming the candidacy of Neurog2 as a causal gene at the Chr 3 QTL" and elsewhere), this isn't true.
They have carried out experiments that show the effects of knocking out this gene, and of expressing Neurog2 in HEK293T cells co-transfected with luciferase plasmids. None of the experiments prove that Neurog2 is the causal gene, or that the E-box variant in the 5'UTR of Isl1 is the causative variant at the chromosome 3 locus. Proving that would require experiments to change causative variants from one strain to the other (e.g. mutating the E-box variant) and demonstrating that the effect seen is via the candidate gene.
If they want to continue to claim they have found the causative gene at the QTL, then they need to provide experimental data to support that claim or rephrase the paper to indicate the genes whose function they investigate are candidates based on their mapping location.

Second, statistical evidence in support of the interaction should be strengthened. It would be helpful if they showed the P-values rather than LODs (which most readers won't understand) but the value they report is borderline (2.86) and they don't report the results of the ANOVA. They refer to Figure 1 E (page 6 line 111) which states "E: The six strains containing the B haplotype at each locus (B/B) have significantly greater numbers of horizontal cells than in the other three groups ($p < 0.001$). A/A is also significantly different from A/B ($p = 0.04$)." What are the numbers in each group and what was the value of the statistic used? Also I can't see any description or display of the phenotypic distribution. Were there any outliers? Are they sure the interaction isn't an artefact of scaling? Did they transform the data (e.g quantile normalization), and test that?
Is the effect robust to a logarithmic transform? An independent experiment, examining retinal cells in an F2 between B6 and A/J would definitely confirm the interaction, but at the very least a better description of what they did is needed. I'd suggest just run two linear models, one with and

© 2023. Published by The Company of Biologists under the terms of the Creative Commons Attribution License (https://creativecommons.org/licenses/by/4.0/).

one without the interaction and report the ANOVA of the difference in fit. Please include the co-variates (sex etc) so readers can assess the contribution of each to the model
The strongest experimental result is from the double knockout. As they say, “losing Neurog2 upon horizontal cell number is abrogated when Isl1 is also eliminated”. I think that result is good, but I’m not sure the E-box luciferase data helps them. Did they really expect HEK293T cells to mimic the transcriptional profile of retinal cells (a comment on this would be useful)? I guess there is no harm in including these data as long as they remove claims that the results says anything (much) about the QTL.

Reviewer 3

Advance summary and potential significance to field

This paper would offer significant new information about the regulation of horizontal cell number in the retina.

Comments for the author

This is an interesting study from the Reese laboratory. It is based on the data from the Whitney et al. 2011 study that define a significant QTL on chromosome 13 which modulates the number of horizontal cells in the mouse retina. Further analysis of these data identified a potentially interacting locus on chromosome 3. The analysis of potential candidate genes within the chromosome 3 locus identified one that would potentially interact with the E-Box identified in the original Whitney study. The authors go on to offer strong evidence that these two genes are interacting and that they are involved in modulating horizontal cell number in the mouse. Unfortunately, the overall enthusiasm for the manuscript is affected by the fact that the peak on chromosome 3 by composite interval mapping does not reach the significance level;
however, this is not unexpected for this kind of analysis. Rarely do peaks revealed by composite mapping reach a significant level. The authors should make this clear and also include a justification for working on the chromosome 3 peak to define interacting genetic elements. Without a clear argument that the study demonstrates the regulation of horizontal cell number by an interaction of Neurog2 and Lsl1, the paper loses its significance and does not provide an overall contribution to science.

The figures for this paper were particularly suited to the study, and added to the understanding of the reader of the complicated process of defining QTLs as well as candidate genes.

Specific Comments:

Line 102, The authors should provide additional information as to the nature of composite interval mapping, what it is and how it works.

Line 104, The authors should clearly state it is a suggestive ($p > 0.67$ and < 0.05) peak on Chr 3 with a LOD score of 2.86. Then the authors should make a statement like, “Although this peak did not reach statistical significance ($p < 0.05$), it did encompass a number of relevant candidate genes.”

Figure legend 1. In line 1 the authors say there was a significant peak on Chr 3, while the figure shows the peak on Chr 13.

Lines 271 and 281, The authors use the word suggest or suggesting to come to conclusions about the interactions of Neurog2 and Isl1 in controlling cell number. This statement is weak and does not affirm the title of the manuscript. If the authors have proven that the interaction of Neurog2 and Lsl1 regulate horizontal cell number, then they should not say the results suggest that Neurog2 and Lsl1 regulate horizontal cell number.

© 2023. Published by The Company of Biologists under the terms of the Creative Commons Attribution License (https://creativecommons.org/licenses/by/4.0/).

First revision

Author response to reviewers' comments

We thank all three reviewers for their thoughtful appraisals of the manuscript and constructive comments. Their criticisms do not always coincide, but we have tried to amend the manuscript accordingly, in response to nearly all of their points. All changes to the manuscript are highlighted in red. We believe the resubmission is far stronger as a consequence, and hope the reviewers come to agree that the sum total of the studies therein provides a compelling contribution for understanding the genetic regulation of horizontal cell number.

Reviewer 1 Advance Summary and Potential Significance to Field:

In this paper, the authors investigate the genetic underpinning of the variations of retinal horizontal cell numbers Ammon different mouse strains. Extending from their previous finding identifying that SNPs in the Isl1 gene locus are significantly associated with horizontal cell number variations, in the current study the authors identify the locus of the Neurogenin2 gene is also associated with this trait. Further, the authors identify an epistatic relationship between Neurogenin2 and Isl1; Neurogenin2 represses Isl1 to overcome the negative effect of Isl1 on horizontal cell numbers via an E box present in one haplotype. They show supportive evidence of the epigenetic relationship by analyzing single and double knockout mice and reporter assays. This is an interesting study overall, addressing an important issue regarding how cell numbers are controlled in the retina. The data are clear and convincing and the paper should be of interest to readers of Development.

Reviewer 1 Comments for the Author:

Some of the critiques I have are detailed below:

1. It is clear that Neurogenin2 and Isl1 are not responsible for all the differences between the two stains studies in horizontal cell numbers, as the differences revealed by the knockouts are much smaller. Other variations may be at play too. The authors should make this clear in the discussion.

Some of this difference may of course arise from the fact that these conditional knockout retinas do not exhibit complete cre-mediated recombination. But as horizontal cell number is a polygenic trait that must be influenced by multiple other genomic loci in addition to the two that we have identified (because the variation in cell number across the 26 RI strains is graded, shown in figure 1C top right, rather than exhibiting just a few step-like changes), the effects attributed to these two loci would not be expected to account for the entire difference across the strains, as the reviewer points out. We now mention both these factors at the end of the first paragraph in the Discussion.

2. The significance of the E box in the Isl1 5' UTR in the epistasis between Neurogenin2 and Isl1 is not fully established, although results from the reporter assay are supportive. It would be more convincing if the authors could show that in the haplotype not carrying the E box, knockout of Neurogenin2 makes no difference as in the case when Isl1 is knocked out.

The proposed experiment would require extensive breeding to backcross the floxed *Neurog2* allele as well as the *Chx10-cre* allele onto the A/J genetic background. Rather, we adopted the *in vitro* strategy to show the genetic interaction's dependency upon the extra E-box, which we believe is convincing. But we see with hindsight that we have exceeded the conclusions that can be drawn about the SNP-dependent interaction occurring *in vivo* modulating horizontal cell number, a criticism also raised by the other reviewers. We have now modified the text in response.

3. How the variations in the Neurogenin2 locus affect its function is vague. More discussion is desired.

© 2023. Published by The Company of Biologists under the terms of the Creative Commons Attribution License (https://creativecommons.org/licenses/by/4.0/).

We now provide a more nuanced consideration of these variants for modulating *Neurog2* expression, including our strategy to identify them, and in turn describing two particularly strong candidate regulatory SNPs indicated in figure 2A, now discussed in the text.

Reviewer 2 Advance Summary and Potential Significance to Field:

This paper describes the mapping of a locus that influences the number of retinal horizontal cells. The authors have previously identified a gene, Is1, on chromosome 1, and now map a second locus on chromosome 13 that contains Neurog2. They present evidence from knockouts and luciferase reporter assays to demonstrate the involvement of this gene also in cell number variation.

Reviewer 2 Comments for the Author:

First, despite claims that they have identified the gene underlying the chr3 QTL (e.g. Page 8 line 166 “confirming the candidacy of Neurog2 as a causal gene at the Chr 3 QTL” and elsewhere), this isn’t true. They have carried out experiments that show the effects of knocking out this gene, and of expressing Neurog2 in HEK293T cells co- transfected with luciferase plasmids. None of the experiments prove that Neurog2 is the causal gene, or that the E-box variant in the 5′UTR of Isl1 is the causative variant at the chromosome 3 locus. Proving that would require experiments to change causative variants from one strain to the other (e.g. mutating the E-box variant) and demonstrating that the effect seen is via the candidate gene. If they want to continue to claim they have found the causative gene at the QTL, then they need to provide experimental data to support that claim or rephrase the paper to indicate the genes whose function they investigate are candidates based on their mapping location.

As indicated above, we now recognize that our original claim had exceeded our results by implying we had provided direct evidence for an *in vivo* role for the SNP creating the E-box to permit *Neurog2* to modulate horizontal cell number, and have now edited the manuscript accordingly. We have also carefully reworded the phrase quoted above to clarify that the *Neurog2* CKO results *elevate Neurog2* as a candidate gene at the locus, without claiming that we have proven it.

More generally, what this set of studies has shown is the following: we identified a pair of candidate genes at these two genomic loci (i.e. “based on their mapping location", as the reviewer points out) which, when knocked out, each modulate the very trait that we had used to identify these genomic loci in the first place. We further noted the epistatic interaction between these loci in that mouse strains bearing the *B* haplotype at each of these two loci had significantly greater numbers of horizontal cells than did mouse strains with the three other possible haplotype combinations. Of note, average horizontal cell numbers for those other three groups showed only modest differences between *A/A* with either *A/B* or *B/A* which, when summed together, did not approach the magnitude of the effect when bearing the *B* haplotype at both loci (fig. 1E). We then proposed a plausible interaction between these two particular genes at these respective loci based on one of them being a bHLH transcription factor while the other contains an E-box in the 5’UTR created by an SNP discriminating the two genomes. We in turn confirmed such an interaction modulates gene expression *in vitro*, provided the critical SNP is introduced to yield the E-box. And finally, we verified in the double CKO retina that the effect of knocking out *Neurog2* upon horizontal cell number is abolished when *Isl1* is also eliminated. The latter, we believe, does indeed show *in vivo* evidence for a genetic interaction between these genes (if not the role of the SNP) to modulate horizontal cell number.

We have now attempted a judicious editing of the entire manuscript to make clearer this distinction between our having shown an interaction between these genes regulating horizontal cell number (in the CKO and DCKO analyses) versus what we speculate the significance of the E-box dependency shown *in vitro* should mean for retinal development.

Second, statistical evidence in support of the interaction should be strengthened. It would be helpful if they showed the P-values rather than LODs (which most readers won’t understand) but the value they report is borderline (2.86) and they don’t report the results of the ANOVA.

© 2023. Published by The Company of Biologists under the terms of the Creative Commons Attribution License (https://creativecommons.org/licenses/by/4.0/).

Such p values can be confusing, as they are not adjusted for multiple testing, and corrections such as the Bonferroni are excessively conservative (leading the false negatives) due to the fact that the multiple tests are not independent because of linkage disequilibrium. We prefer to retain the convention of using LOD scores as they are common in such QTL mapping studies (and apparently had not been an issue for the other reviewers), but we have now modified the text to make clearer what they mean, and how they are judged for significance via permutation testing.

They refer to Figure 1 E (page 6 line 111) which states "E: The six strains containing the B haplotype at each locus (B/B) have significantly greater numbers of horizontal cells than in the other three groups ($p < 0.001$). A/A is also significantly different from A/B ($p = 0.04$)." What are the numbers in each group and what was the value of the statistic used? Also I can't see any description or display of the phenotypic distribution. Were there any outliers? Are they sure the interaction isn't an artefact of scaling? Did they transform the data (e.g quantile normalization), and test that? Is the effect robust to a logarithmic transform? An independent experiment, examining retinal cells in an F2 between B6 and A/J would definitely confirm the interaction, but at the very least a better description of what they did is needed. I'd suggest just run two linear models, one with and one without the interaction and report the ANOVA of the difference in fit. Please include the co-variates (sex etc) so readers can assess the contribution of each to the model.

We have not attempted the F2 experiment proposed by the reviewer, because of the amount of time it would require to breed, phenotype and genotype such a large number of mice. But we appreciate the reviewer's suggestion to present a more rigorous statistical analysis of the interaction between the two QTL, using a General Linear Model in SPSS. We believe the following additions and clarifications to the text should address the reviewer's concerns.

First, we confirmed that the distribution of phenotypes across the recombinant inbred strains included no outliers for horizontal cell number. One strain, BXA5, was identified as an outlier for *Isl1* expression across the strains; however, removal of this strain did not meaningfully alter the results of the subsequent statistical analyses.

We have now also tested for the effect of the covariate of age on horizontal cell number, finding no significant contribution to the General Linear Model, described below ($p = 0.64$), and thus excluding it as a factor moving forward; similarly, we found no effect of age on *Isl1* expression across the strains ($p = 0.83$). Additionally, all mice used in the mapping study were female. We have now indicated all of this in the Methods. We also now include the X-axis scale for cell number in figure 1D, displaying the phenotypic variation across all strains, and indicate the n's per group at the base of figures 1E and F, where the individual circles display the phenotypic distribution per group.

Second, we have now adopted a two-way ANOVA instead of the original one-way ANOVA, at the reviewer's suggestion, to assess the presence of a statistical interaction. The ANOVA revealed significant main effects of each QTL (Chr 3, $p < 0.001$; Chr 13, $p < 0.001$), as well as a significant interaction between the two QTL (Chr 3 * Chr 13, $p = 0.006$). Pairwise comparisons revealed that when a strain has the *A* haplotype on Chr 13 (i.e. no E- BOX), the haplotype on Chr 3 does not significantly affect cell number ($p = 0.31$), but when a strain has the *B* haplotype on Chr 13 (i.e. E-BOX), the haplotype on Chr 3 has a significant effect on cell number ($p < 0.001$).
These results corroborate our original findings. We now substitute the results of this two-way ANOVA for the original one-way ANOVA, addressing it in the Methods, Results and in figure 1.

Third, we normalized our data as suggested by the reviewer, performing both a logarithmic and quantile transformation to assess any effects of scaling. The significant main effects and significant interaction in the two- way ANOVA were maintained using both transformations (now indicated in the text), indicating that the findings with the original raw data were robust, shown here:

© 2023. Published by The Company of Biologists under the terms of the Creative Commons Attribution License (https://creativecommons.org/licenses/by/4.0/).

No Transformation

	df	F	Sig.
Chr 3	1	19.822	< 0.001
Chr 13	1	45.752	< 0.001
Chr 3*Chr 13	1	9.127	0.006

Chr 13	Chr 3	Sig.
A	*A* vs *B*	0.314
B	*A* vs *B*	< 0.001

Log2 Transformation

	df	F	Sig.
Chr 3	1	17.619	< 0.001
Chr 13	1	43.378	< 0.001
Chr 3*Chr 13	1	7.585	0.012

Chr 13	Chr 3	Sig.
A	*A* vs *B*	0.310
B	*A* vs *B*	< 0.001

Quantile Transformation

	df	F	Sig.
Chr 3	1	6.998	0.015
Chr 13	1	24.167	< 0.001
Chr 3*Chr 13	1	4.894	0.038

Chr 13	Chr 3	Sig.
A	*A* vs *B*	0.758
B	*A* vs *B*	0.003

Finally, we assessed the contribution of the interaction to the linear model, by comparing the "fit" of the model with and without the interaction factor (Chr 3 * Chr 13). The adjusted R^2 for the full model including the interaction was 0.751, while the adjusted R^2 of the model lacking the interaction was 0.663, indicating that the interaction model fits the data better, accounting for 75% of the variance observed in HC number across the strains. Likewise, we found that the model with the interaction accounts for 41% of the variance in *Isl1* expression across the strains, while the model lacking the interaction does not fit the data well at all, accounting for only 7% of the variance. We have now indicated these differences in fit in the legend to figure 1.

In summary, we checked for outliers, we checked for robustness by transforming the data, we checked the contribution of age and sex, and we checked for the contribution of the interaction upon the fit of the data. All evidence reinforces the significance of the interaction between the two QTLs. Doubtless the case is far stronger for all these efforts, for which we thank the reviewer.

We would also like to note that in the process of revisiting *all* of our statistical analyses, we discovered an oversight in the one-way ANOVA shown in panel 4D, mistaking the p value to be 0.3 rather than 0.03, which post-hoc testing confirmed to arise between the x1 versus x2 conditions. None of the NEUROG2 conditions, x1, x2 and x3, differed from the control condition, unlike in panel 4C, in the presence of the E-box. We now address this in the Results section.

The strongest experimental result is from the double knockout. As they say, "losing Neurog2 upon horizontal cell number is abrogated when Isl1 is also eliminated". I think that result is good, but I'm not sure the E-box luciferase data helps them. Did they really expect HEK293T cells to mimic the transcriptional profile of retinal cells (a comment on this would be useful)? I guess there is no harm in including these data as long as they remove claims that the results says anything (much) about the QTL.

We did not imagine the transcriptional profile of retinal cells would be present in HEK293T cells. Rather, we wanted to assess the bare minimum of bringing together NEUROG2 in the presence of the extra E-box in *Isl1* versus in its absence, in order to create the critical conditions that we speculate could be instrumental in the modulation of horizontal cell number *in vivo*. We now make the rationale behind this experiment clearer in the Results section, as requested by the reviewer.

Reviewer 3 Advance Summary and Potential Significance to Field:

This paper would offer significant new information about the regulation of horizontal cell number in the retina.

Reviewer 3 Comments for the Author:

This is an interesting study from the Reese laboratory. It is based on the data from the Whitney et al. 2011 study that define a significant QTL on chromosome 13 which modulates the number of horizontal cells in the mouse retina. Further analysis of these data identified a potentially interacting locus on chromosome 3. The analysis of potential candidate genes within the chromosome 3 locus identified one that would potentially interact with the E-Box identified in the

© 2023. Published by The Company of Biologists under the terms of the Creative Commons Attribution License (https://creativecommons.org/licenses/by/4.0/).

original Whitney study. The authors go on to offer strong evidence that these two genes are interacting and that they are involved in modulating horizontal cell number in the mouse. Unfortunately, the overall enthusiasm for the manuscript is affected by the fact that the peak on chromosome 3 by composite interval mapping does not reach the significance level; however, this is not unexpected for this kind of analysis. Rarely do peaks revealed by composite mapping reach a significant level. The authors should make this clear and also include a justification for working on the chromosome 3 peak to define interacting genetic elements.
Without a clear argument that the study demonstrates the regulation of horizontal cell number by an interaction of Neurog2 and Lsl1, the paper loses its significance and does not provide an overall contribution to science.

We now draw greater attention to the fact that the Chr 3 QTL does not cross the significant threshold, and that this is not uncommon following composite interval mapping, all as requested or noted by the reviewer. But to assure the reviewer of the presence of this locus, we have now confirmed it using GEMMA (the Genome-wide Efficient Mixed Model Association mapping option in GeneNetwork). While this option does not permit controlling for variation at one locus, as above for composite interval mapping, it confirms the presence of both loci on Chrs 3 and 13, increasing the relative magnitude of the Chr 3 locus while reducing the relative magnitude of the Chr 13 locus, shown here:

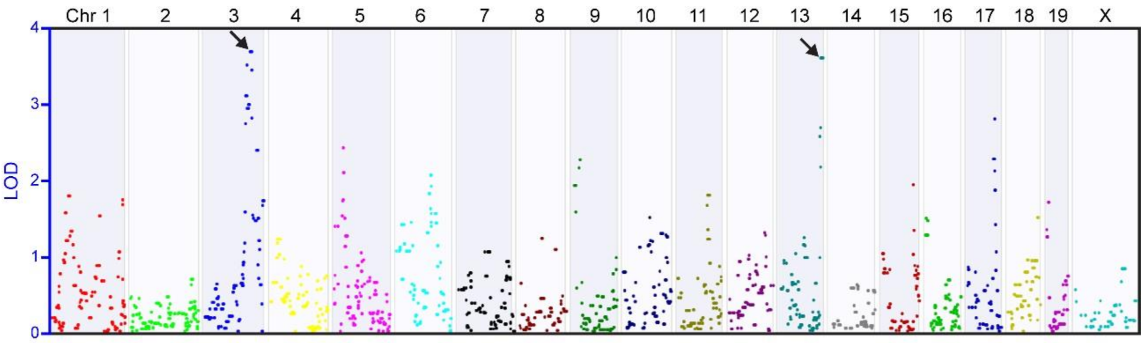

Rather than belaboring the relative strengths and weaknesses of the different mapping approaches, we regard both as focusing our attention on these two loci. But the strongest basis for working on the Chr 3 locus, *without doubt*, arises from the demonstration of an epistatic interaction between this very locus with the locus on Chr 13, now having shown it to be a statistically significant interaction that fits a large amount (75%) of the variance in horizontal cell number, described above. That a gene identified at this Chr 3 locus modulates horizontal cell number when knocked out is further assurance, if not proof, that the suggestive locus is meaningful

Further validation of the locus comes from the significant interaction detected between the haplotypes at the two QTL accounting for 41% of the variance in *Isl1* expression across the strains. Of note, *Isl1* expression is reduced in strains with the novel E-box, *but only* when in the presence of the *B* haplotype at the Chr 3 locus. We now indicate all of this in the revised manuscript.

We believe we have already laid out what is novel and noteworthy about the present QTL study in response to the second reviewer's first paragraph. But with regard to the final point in the above paragraph, while we have not shown definitive *in vivo* proof that the SNP creating the E-box gates the effectiveness of NEUROG2 in modulating horizontal cell number via its control upon *Isl1* expression, we do believe the CKO and DCKO analyses "demonstrates the regulation of horizontal cell number by an interaction of Neurog2 and Isl1" *in vivo*. We have, consequently, now been careful about when we use "suggest" versus "indicate" throughout the manuscript.

The figures for this paper were particularly suited to the study, and added to the understanding of the reader of the complicated process of defining QTLs as well as candidate genes.

© 2023. Published by The Company of Biologists under the terms of the Creative Commons Attribution License (https://creativecommons.org/licenses/by/4.0/).

Specific Comments:

Line 102, The authors should provide additional information as to the nature of composite interval mapping, what it is and how it works.

We have now provided further clarification on this.

Line 104, The authors should clearly state it is a suggestive ($p > 0.67$ and < 0.05) peak on Chr 3 with a LOD score of 2.86. Then the authors should make a statement like, "Although this peak did not reach statistical significance ($p < 0.05$), it did encompass a number of relevant candidate genes."

We have now modified the text in the Results section to clarify the p-value issue raised here.

Figure legend 1. In line 1 the authors say there was a significant peak on Chr 3, while the figure shows the peak on Chr 13.

We have now corrected this error.

Lines 271 and 281, The authors use the word suggest or suggesting to come to conclusions about the interactions of Neurog2 and Isl1 in controlling cell number. This statement is weak and does not affirm the title of the manuscript. If the authors have proven that the interaction of Neurog2 and Lsl1 regulate horizontal cell number, then they should not say the results suggest that Neurog2 and Lsl1 regulate horizontal cell number.

We have now carefully edited the text to discriminate our claims for proving the interaction between these two genes (*Neurog2* and *Isl1*) *in vivo* (demonstrated in the CKO and DCKO retinas), versus our speculation that the *in vitro* SNP-dependency of this interaction (demonstrated by the luciferase assays) is responsible for the variation in horizontal cell number. Our lack of clarity about this distinction in the original manuscript led to our drifting inconsistently between "suggest" versus "indicate".

<u>Second decision letter</u>

MS ID#: DEVELOP/2022/201315

MS TITLE: Neurog2 Regulates Isl1 to Modulate Horizontal Cell Number

AUTHORS: Patrick W Keeley, Pooja S Patel, Matthew S Ryu, and Benjamin E Reese
ARTICLE TYPE: Research Article

I am happy to tell you that your manuscript has been accepted for publication in Development, pending our standard ethics checks.

<u>Reviewer 2</u>

Advance summary and potential significance to field

This paper makes a useful contribution to the literature on genes involved in determining the number of retinal horizontal cells and provides a nice illustration of epistatic effects on that process

Comments for the author

The authors have addressed my concerns

© 2023. Published by The Company of Biologists under the terms of the Creative Commons Attribution License (https://creativecommons.org/licenses/by/4.0/).

Reviewer 3

Advance summary and potential significance to field

The paper describes the mapping of a suggestive locus that influences the number of retinal horizontal cells.
The authors have previously identified a gene, Isl1, on chromosome 3, and now map a second suggestive locus on chromosome 13 using composite mapping methods. They have looked at one gene within that as a modifier of Isl1. The data is suggestive but not definitive.

Comments for the author

In the previous submission of the manuscript the authors identified a suggestive QTL on Chr3 using composite mapping methods. This locus appeared to modulate Neurog2 and horizontal cell number. Within this locus there were 61 protein-coding genes or microRNAs. Of these 61 genes only one was taken forward to test its interaction with Isl1. The initial review of the manuscript criticized the lack of definitive proof of the interactions and the speculative nature of the findings. The argument for the identification of Neurog2 as the gene interacting with Isl1 made in the response to the review of the manuscript as well as the modifications made in the manuscript do not provide definitive proof for the interaction. Unfortunately, they have not provided experimental data to support their claim and the resulting modifications only lead to a conclusion of a potential candidate with suggestive interaction.

© 2023. Published by The Company of Biologists under the terms of the Creative Commons Attribution License (https://creativecommons.org/licenses/by/4.0/).
